# PREVENT and PCE Models for Estimating ASCVD Risk Stratified by Statin Exposure

**DOI:** 10.1001/jamanetworkopen.2025.32164

**Published:** 2025-09-16

**Authors:** Ming-Sum Lee, James Onwuzurike, Yi-Lin Wu, Darryl E. Palmer-Toy, Jaejin An, Wansu Chen

**Affiliations:** 1 Department of Cardiology, Kaiser Permanente Los Angeles Medical Center, Los Angeles, California; 2 Department of Research and Evaluation, Kaiser Permanente Southern California, Pasadena; 3 SCPMG Regional Reference Laboratories, Kaiser Permanente Southern California, North Hollywood

## Abstract

**Importance:**

The Predicting Risk of Cardiovascular Disease Events (PREVENT) equations are an updated model developed to improve on the Pooled Cohort Equation (PCE) for estimating 10-year atherosclerotic cardiovascular disease (ASCVD) risk. These equations facilitate patient-clinician discussions on initiating statin therapy and are used to estimate risk without treatment. However, statin exposure during follow-up was not fully accounted for in the development of these equations.

**Objective:**

To assess the performance of the PCE and PREVENT equations in estimating ASCVD, accounting for statin exposure during follow-up.

**Design, Setting, and Participants:**

This retrospective cohort study included adults from an integrated health care system with 10-year follow-up data. Adults without diabetes or ASCVD were identified in 2013 and followed-up through December 31, 2023, with analyses performed in January 2025.

**Main Outcomes and Measures:**

The primary outcome was incident ASCVD. Estimated risks from PCE and PREVENT equations were compared with observed risks, with discrimination assessed via C statistics. The performance of these equations was evaluated in patient populations stratified by statin exposure during follow-up.

**Results:**

Among 193 885 adults (median [IQR] age, 55 [48-63] years; 113 400 [58.5%] women), 6528 experienced an ASCVD event. The C statistic was 0.725 (95% CI, 0.719-0.731) for PCE and 0.723 (95% CI, 0.716-0.729) for PREVENT. In the overall population, regardless of statin exposure, the observed 10-year ASCVD risk was lower than estimated by PCE: 3.6% for individuals with estimated risk of 5% to less than 7.5%, 4.5% for those with estimated risk of 7.5% to less than 10%, and 8.0% for those with estimated risk of 10% or greater. The observed risk more closely aligned with the estimated risk from PREVENT: 5.2% for individuals with estimated risk of 5% to 7.5%, 8.1% for those with estimated risk 7.5% to less than 10%, and 11.6% for those with estimated risk of 10% or greater. In contrast, among patients not exposed to statin therapy during follow-up, PREVENT underestimated risk: observed risk was 8.2% for individuals with estimated risk of 5% to less than 7.5%, and 13.5% for those with estimated risk of 7.5% to less than 10%, while PCE-estimated risk more closely approximated the observed risk.

**Conclusions and Relevance:**

In this retrospective cohort study, the PREVENT model underestimated risk in patients not treated with statins, whereas the PCE estimates more closely reflected what a patient’s risk would be without statin therapy.

## Key Points

**Question:**

How do the Predicting Risk of Cardiovascular Disease Events (PREVENT) and Pooled Cohort Equation (PCE) models perform in estimating 10-year atherosclerotic cardiovascular disease (ASCVD) risk when accounting for statin therapy exposure?

**Findings:**

In this cohort study including 193 885 patients, among patients who were not exposed to statins during follow-up, PREVENT underestimated risk, whereas PCE provided estimates that more closely matched observed ASCVD risk.

**Meaning:**

This cohort study found that compared with the PREVENT estimates, PCE estimates more closely reflected what a patient’s ASCVD risk would be without statin therapy.

## Teaser

This cohort study assesses the calibration and discrimination of the Pooled Cohort Equation (PCE) and Predicting Risk of Cardiovascular Disease Events (PREVENT) models in estimating 10-year risk of atherosclerotic cardiovascular disease (ASCVD), accounting for statin exposure during follow-up.

## Introduction

Cardiovascular diseases are responsible for a substantial burden of morbidity and mortality.^1^ Accurate risk assessment tools are essential to appropriately guide interventions. The American College of Cardiology/American Heart Association Pooled Cohort Equations (PCE) are endorsed by current national guidelines for estimating 10-year rates of atherosclerotic cardiovascular disease (ASCVD) events.^2,3^ The pooled cohort equations were derived in the 1990s, based on cohort studies that included predominantly Black and White participants. Concerns have been raised regarding the generalizability of PCE in contemporary and more diverse populations.^4,5,6^

In response to these limitations, the American Heart Association introduced a new risk prediction model in 2023: the Predicting Risk of Cardiovascular Disease Events (PREVENT) calculator, an updated cardiovascular risk assessment model developed from a more contemporary and racially diverse cohort.^7^ Importantly, the model reflects a shift away from race-based clinical algorithms by removing race as a predictor. Instead, it replaces race with the Social Deprivation Index, a zip code–based proxy for social determinants of health.^8^ Built within a cardiovascular-kidney-metabolic health framework, the calculator takes kidney function and body mass index (BMI; calculated as weight in kilograms divided by height in meters squared) into account.^7^

While PREVENT represents a meaningful step forward conceptually, its performance in large, unselected populations has not been comprehensively evaluated. Notably, prior evaluation has not consistently accounted for whether patients were exposed to statin therapy during the follow-up period. This is important because risk prediction models are designed to estimate baseline cardiovascular risk without treatment. If a model is developed and validated in a population where many individuals later receive therapy, the predicted event rates may be artificially lower due to treatment effects. To be clinically useful, a risk model should accurately predict risk in untreated individuals.

The objective of this study was to evaluate and compare the performance of the PREVENT and PCE models in a large US population with a full 10-year follow-up period. A strength of this analysis is its ability to account for statin exposure using detailed pharmacy records from an integrated health care system. By comparing observed ASCVD incidence with estimated risk in statin-treated and untreated subgroups, this study provides an assessment of the validity of 2 major cardiovascular risk prediction models, informing their optimal use in contemporary, diverse populations.

## Methods

This cohort study was approved by the Kaiser Permanente Southern California (KPSC) institutional review board with a waiver of informed consent based on 45 CFR §46. This study is reported in accordance with the Strengthening the Reporting of Observational Studies in Epidemiology (STROBE) reporting guideline.

### Study Design and Data Source

This is a retrospective, population-based cohort study that included members of KPSC, a large integrated health care delivery system providing comprehensive inpatient and outpatient services to more than 4.8 million individuals.^9^ KPSC maintains robust, prospectively collected electronic databases encompassing demographics, pharmacy records, laboratory results, and health care utilization data from both ambulatory and inpatient settings.

### Study Population

The study included KPSC health plan enrollees between January 1, 2013, and December 31, 2013, who were aged 40 to 75 years with a low-density lipoprotein cholesterol (LDL-C) level between 70 and 189 mg/dL (to convert to millimoles per liter, multiply by 0.0259) in 2013. The date of the first qualifying lipid panel measurement was used as the index date. The following exclusion criteria were applied: dialysis, hospice care, baseline history of ASCVD (myocardial infarction, ischemic stroke, peripheral artery disease, history of coronary artery bypass surgery or percutaneous coronary intervention), diabetes (given existing guideline-based statin indication), dispensing of statins within 2 years before the index date, fewer than 12 months of continuous health plan enrollment before the index dates (to ensure complete baseline data), and fewer than 10 years of complete membership and follow-up (except in cases where the patient died during follow-up).

Patients with missing total cholesterol, high-density lipoprotein cholesterol, blood pressure, BMI, or creatinine within 1 year of the index date were excluded, as these variables were required for estimating ASCVD risk using the PCE or PREVENT equations. Since PCE and PREVENT were designed to be used with total cholesterol between 130 and 320 mg/dL, high-density lipoprotein cholesterol between 20 and 200 mg/dL, BMI between 13.5 and 39.9, and estimated glomerular filtration rate between 14 and 140 mL/min/1.73 m^2^, patients with values outside of these ranges were excluded from the study.

### Outcomes

Patients were followed-up from the index date until the first occurrence of an ASCVD event, death, disenrollment from the health plan, or the study end date (December 31, 2023). The primary outcome was incident ASCVD, defined as acute myocardial infarction, fatal or nonfatal stroke, or coronary heart disease death. Myocardial infarction and stroke were identified using the principal discharge diagnosis from hospital records and billing claims databases, using *International Classification of Diseases, Ninth Revision (ICD-9) *and *International Statistical Classification of Diseases and Related Health Problems, Tenth Revision (ICD-10) *codes (myocardial infarction: 410, I21, I22; stroke: 431, 432, 433.x1, 434.x1, I61, I62, I63).^8^ Mortality was extracted from a mortality data mart that includes death information from insurance plan administrative records, Social Security Administration death master files, and hospital death records. Cause of death was identified using *ICD-9* and *ICD-10* codes.

### ASCVD Risk Estimation

The 10-year estimated ASCVD risk was calculated for each patient using both the PCE and the PREVENT equations.^2,8^ The PREVENT base model incorporates traditional cardiovascular risk factors and kidney function as key risk factors, while the PREVENT full model includes additional variables (including urine albumin-to-creatinine ratio, hemoglobin A_1c_, and the Social Deprivation Index). The primary analyses focused on using the PREVENT base model in estimating ASCVD risk. Patients were stratified into 4 risk groups based on guideline-recommended thresholds for statin initiation and intensification: less than 5%, 5% to less than 7.5%, 7.5% to less than 10%, and 10% and above, based on estimated risk.

### Covariates

Patient demographic characteristics were collected from electronic health records. Race and ethnicity were self-reported and categorized as Asian, Black, Hispanic, White, and other (comprising unknown or declined and patients indicating their race as multiple or other). Medical comorbidities were identified using *ICD-9* and *ICD-10* codes. Smoking status was self-reported.

Statin exposure was ascertained using outpatient pharmacy dispensing records. Patients were considered exposed to statin therapy during the follow-up period if they obtained at least 1 dispensing of statin between the index date and the end of follow-up (ASCVD event, death, disenrollment from the health plan, or end of study follow-up). High adherence to statin therapy during the follow-up period was defined as a proportion of days covered of 80% or higher.^10,11^

### Statistical Analysis

Continuous variables were reported in medians with IQRs or means with SDs. Categorical variables were presented as counts and percentages. Baseline characteristics were compared using analysis of variance for continuous variables and χ^2^ tests for categorical variables. The observed 10-year incidence of ASCVD events was calculated as the proportion of patients with at least 1 ASCVD event during follow-up. Calibration was assessed by comparing observed and estimated risks across risk strata using calibration plots.^6^ Calibration slope was calculated. Discrimination was evaluated using C statistics, with C statistics above 0.7 considered a reasonable model. Kaplan-Meier curves were generated to display event-free survival and compared using the log-rank test. A sensitivity analysis was performed by applying Fine-Gray subdistribution hazard models to estimate the adjusted 10-year risk, considering death from noncoronary heart disease as a competing risk.^12,13^ All statistical analyses were 2-sided, with a significance threshold of *P* < .05. Analyses were performed using Stata version 17/MP 17.0 (StataCorp) and SAS version 9.4 (SAS Institute) in January 2025.

## Results

### Study Population

A total of 193 885 adults between ages 40 and 75 years met the inclusion criteria (eFigure 1 in Supplement 1). The median (IQR) age of the cohort was 55 (48-63) years, and 113 400 (58.5%) were women. The population was racially and ethnically diverse, with 27 496 participants (14.2%) self-identifying as Asian, 19 667 participants (10.1%) as Black, 64 179 participants (33.1%) as Hispanic, and 79 031 participants (40.8%) as White. Chronic kidney disease was present in 7028 participants (3.6%), 11 250 participants (5.8%) were active smokers, and 60 079 participants (31.0%) were receiving antihypertensive treatment at baseline (Table 1). The mean (SD) BMI was 28.0 (4.7), and the mean (SD) and estimated glomerular filtration rate was 92 (16) mL/min/1.73 m^2^.

**Table 1.** Baseline Characteristics Stratified by ASCVD Risk Category Derived From the PREVENT Base Model

Characteristic	Patients by PREVENT base equation 10-y estimated risk of atherosclerotic cardiovascular disease, No. (%)					*P* value^a^
	Overall (N = 193 885)	<5% (n = 141 979)	5% to <7.5% (n = 28 317)	7.5% to <10% (n = 14 509)	≥10% (n = 9080)	
Age, y						
Mean (SD)	55.5 (9.3)	51.6 (7.1)	63.8 (5.4)	67.8 (4.7)	70.8 (4.1)	<.001
Median (IQR)	55 (48-63)	51 (46-57)	64 (61-67)	68 (65-71)	72 (69-74)	<.001
Sex						
Women	113 400 (58.5)	90 629 (63.8)	14 185 (50.1)	6214 (42.8)	2372 (26.1)	<.001
Men	80 485 (41.5)	51 350 (36.2)	14 132 (49.9)	8295 (57.2)	6708 (73.9)	
Race and Ethnicity						
Asian	27 496 (14.2)	21 397 (15.1)	3477 (12.3)	1644 (11.3)	978 (10.8)	<.001
Black	19 667 (10.1)	14 055 (9.9)	3026 (10.7)	1569 (10.8)	1017 (11.2)	
Hispanic	64 179 (33.1)	51 461 (36.3)	7040 (24.9)	3443 (23.7)	2235 (24.6)	
White	79 031 (40.8)	52 304 (34.8)	14 335 (50.6)	7665 (52.8)	4727 (52.1)	
Other^b^	3512 (1.8)	2762 (2.0)	439 (1.6)	188 (1.3)	123 (1.4)	
Hypertension	62 046 (32.0)	32 258 (22.7)	14 100 (49.8)	8982 (61.9)	6706 (73.9)	<.001
Chronic kidney disease	7028 (3.6)	2769 (2.0)	1663 (5.9)	1370 (9.4)	1226 (13.5)	
Systolic blood pressure, mean (SD) mm Hg	126 (16)	122 (15)	133 (16)	137 (17)	142 (19)	<.001
Total cholesterol, mean (SD), mg/dL	203 (32)	202 (31)	206 (32)	203 (32)	202 (32)	<.001
HDL, mean (SD), mg/dL	54 (14)	55 (14)	53 (14)	51 (13)	47 (11)	<.001
LDL, mean (SD), mg/dL	123 (26)	123 (26)	126 (26)	125 (26)	125 (27)	<.001
BMI, mean (SD)	28.0 (4.7)	27.9 (4.7)	28.2 (4.6)	28.2 (4.4)	28.4 (4.3)	<.001
Current smoking	11 250 (5.8)	6251 (4.4)	2408 (8.5)	1376 (9.5)	1215 (13.4)	<.001
Antihypertensive treatment	60 079 (31.0)	30 246 (21.3)	13 768 (48.6)	9025 (62.2)	7040 (77.5)	<.001
eGFR, mean (SD), mL/min per 1.72 m^2^	92 (16)	95 (15)	85 (15)	81 (15)	77 (16)	<.001
uACR, median (IQR), mg/g	5.8 (3.2-13.7)	5.3 (3-12.2)	6.2 (3.4-14.1)	6.6 (3.5-16.2)	8.1 (4.1-22.3)	<.001
Glycated hemoglobin, mean (SD), %	5.8 (0.6)	5.8 (0.6)	5.9 (0.6)	5.9 (0.6)	6.0 (0.7)	<.001
Social Deprivation Index decile (1-10)	5.1 (2.9)	5.2 (2.9)	4.9 (2.8)	5.0 (2.9)	5.1 (2.9)	<.001

Abbreviations: ASCVD, atherosclerotic cardiovascular disease; BMI, body mass index (calculated as weight in kilograms divided by height in meters squared); eGFR, estimated glomerular filtration rate; HDL, high-density lipoprotein; LDL, low-density lipoprotein; PREVENT, Predicting Risk of Cardiovascular Disease Events; uACR, urine albumin-to-creatinine-ratio.

SI conversion factors: To convert cholesterol to millimoles per liter, multiply by 0.0259; glycated hemoglobin to proportion of total hemoglobin, multiply by 0.01.

^a^ Analysis of variance for continuous variables and χ^2^ for categorical variables across risk categories.

^b^ Includes multiple, unknown or declined, and patients indicating their race as other.

Patients were stratified into 4 risk groups using the PREVENT base equation: 141 979 patients (73.2%) had a estimated 10-year ASCVD risk of less than 5%, 28 317 patients (14.6%) had a estimated risk between 5% and less than 7.5%, 14 509 patients (7.4%) had a estimated risk between 7.5% and less than 10%, and 9080 patients (4.7%) had a estimated risk of 10% or greater (Table 1). During the follow-up period, 99 327 patients (51.2%) were not exposed to statin therapy, while 94 558 patients (48.8%) filled at least 1 statin prescription. Among patients exposed to statin therapy, 10 658 (11.3%) were exposed to statin therapy for more than 80% of the follow-up period.

### Outcomes

**Figure 1.**
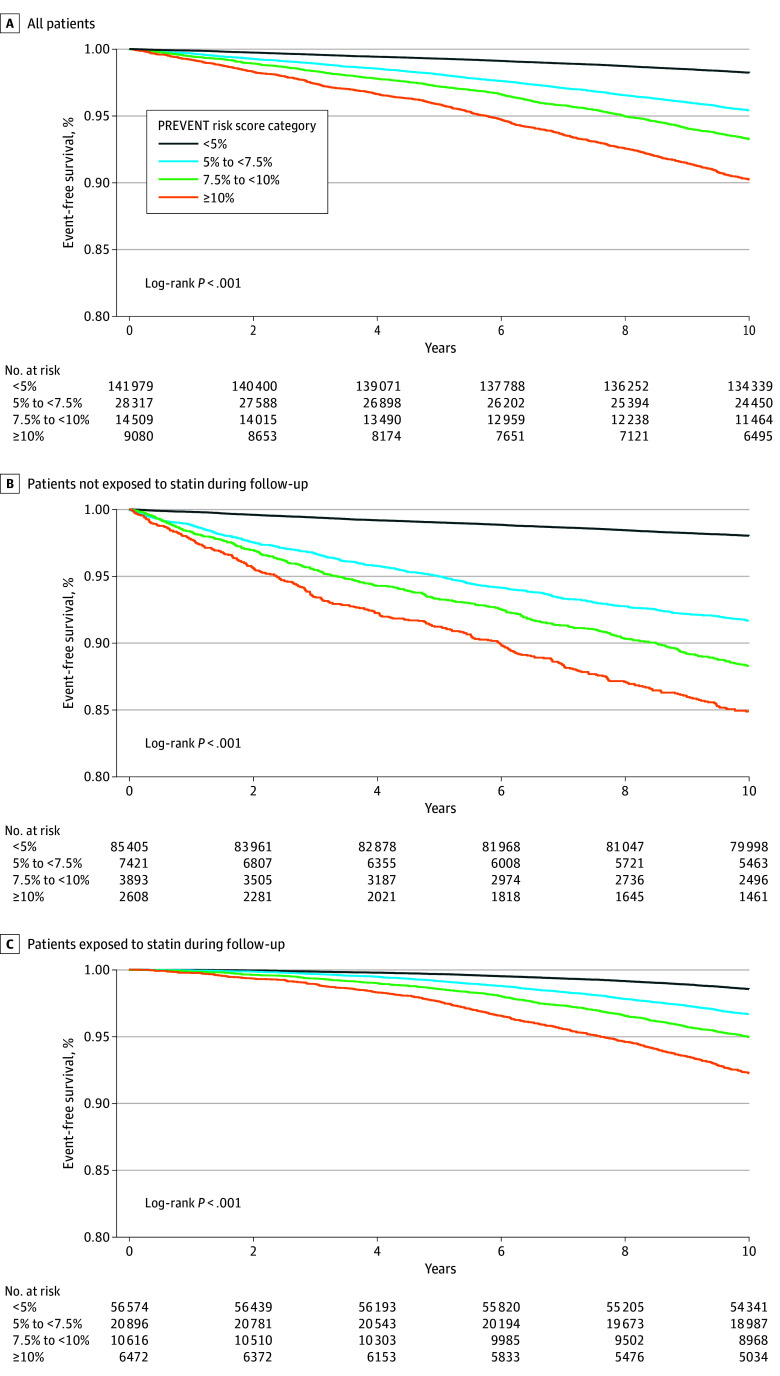
Kaplan-Meier Curves Stratified by Predicting Risk of Cardiovascular Disease Events (PREVENT)

Over the course of the 10-year follow-up, there were 6528 incident ASCVD events. Figure 1 shows the Kaplan-Meier event-free survival curves, stratified by estimated risk of ASCVD at baseline. Higher event-free survival was observed among patients exposed to statin (Figure 1C), with the highest event free-survival observed among individuals exposed to statin therapy for more than 80% of the follow-up period (eFigure 2 in Supplement 1).

### Observed and Estimated Incidence Rates

**Figure 2.**
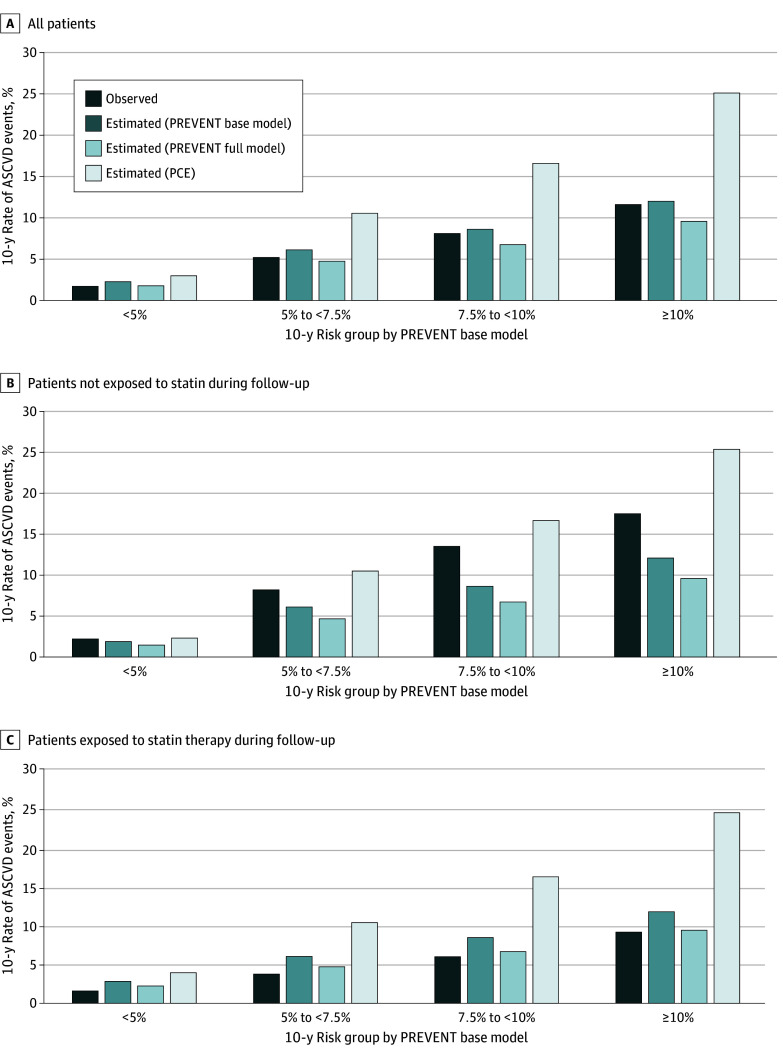
Observed and Estimated Incidence Rates of Atherosclerotic Cardiovascular Events (ASCVD) PCE indicates Pooled Cohort Equation; PREVENT, Predicting Risk of Cardiovascular Disease Events.

Observed and estimated 10-year incidence rates of ASCVD were compared within baseline risk categories (Figure 2). In the overall cohort (not accounting for statin-exposure during follow-up), the PREVENT base model generally yielded estimated risks that were similar to the observed event rates. The PREVENT full model estimated lower estimated risks compared with the base model, while the PCE model consistently yielded higher estimated risks than either version of the PREVENT model: (Figure 2A).

Among patients not exposed to statin therapy during follow-up, observed 10-year ASCVD incidence rates exceeded the estimated risks from both the PREVENT base and full models (Figure 2B). In contrast, among patients exposed to statin therapy, observed event rates closely aligned with the PREVENT full model’s estimated rates while being lower than the rates estimated by the PREVENT base model and PCE (Figure 2C). Notably, patients exposed to statin therapy for more than 80% of the follow-up period experienced substantially lower event rates than estimated by any of the models (eFigure 3 in Supplement 1).

Table 2 shows the estimated incidence rates of ASCVD according to the PREVENT base model and PCE, alongside the corresponding crude observed rates and adjusted observed estimates derived from Fine-Gray subdistribution models. In the overall cohort (not accounting for statin exposure during follow-up), the observed 10-year ASCVD risk was lower than estimated by PCE: 3.6% for individuals with estimated risk of 5% to less than 7.5%, 4.5% for those with estimated risk of 7.5% to less than 10%, and 8.0% for those with estimated risk of 10% or greater. The observed risk more closely aligned with the estimated risk from PREVENT: 5.2% for individuals with estimated risk of 5% to less than 7.5%, 8.1% for those with estimated risk 7.5% to less than 10%, and 11.6% for those with estimated risk of 10% or greater. Among patients who were not exposed to statin therapy during follow-up, observed rates consistently exceeded PREVENT-estimated rates across all risk categories, at 8.2% for individuals with estimated risk of 5% to less than 7.5% and 13.5% for those with estimated risk of 7.5% to less than 10%), while PCE-estimated risk more closely approximated the observed risk.

**Table 2.** Observed and Estimated Incidence Rates of Atherosclerotic Cardiovascular Events Stratified by 10-Year Estimated Risk

Estimated 10-y risk category	Events, No.	10-y Incidence rate per 100 PY			Calibration		Discrimination, C statistic (95% CI)
		Actual observed 10-y, estimate (95% CI)	Adjusted observed, estimate (95% CI)^a^	Estimated	Hosmer-Lemeshow χ^2^	*P* value	
**PREVENT base equations**							
Overall							
<5%	2821	2.0 (1.9-2.1)	1.7 (1.6-1.8)	2.3	298.0	<.001	0.723 (0.716-0.729)
5% to <7.5%	1479	5.2 (5.0-5.5)	4.3 (4.1-4.6)	6.1			
7.5% to <10%	1171	8.1 (7.6-8.5)	6.2 (5.8-6.6)	8.6			
≥10%	1057	11.6 (11.0-12.3)	8.7 (8.2-9.3)	12.0			
No statin during follow-up							
<5%	1907	2.2 (2.1-2.3)	1.9 (1.8-2.0)	1.9	457.2	<.001	0.777 (0.769-0.784)
5% to <7.5%	686	8.2 (8.6-9.9)	7.5 (6.9-8.1)	6.1			
7.5% to <10%	525	13.5 (12.4-14.6)	10.1 (9.2-11.1)	8.6			
≥ 10%	457	17.5 (16.1-19.0)	12.7 (11.5-14.0)	12.1			
Statin during follow-up							
<5%	914	1.6 (1.5-1.7)	1.4 (1.3-1.5)	2.9	66.5	<.001	0.701 (0.691-0.710)
5% to <7.5%	793	3.8 (3.5-4.1)	3.2 (3.0-3.5)	6.1			
7.5% to <10%	646	6.1 (5.6-6.5)	4.8 (4.4-5.2)	8.6			
≥ 10%	600	9.3 (8.6-10.0)	7.1 (6.5-7.8)	12.0			
**PCE equations**							
Overall							
<5%	1759	1.5 (1.4-1.6)	1.3 (1.3-1.4)	2.1	527.0	<.001	0.725 (0.719-0.731)
5% to <7.5%	837	3.6 (3.4-3.9)	3.1 (2.8-3.3)	6.2			
7.5% to <10%	710	4.5 (4.2-4.8)	3.8 (3.5-4.1)	8.7			
≥10%	3222	8.0 (7.7-8.3)	6.3 (6.1-6.5)	16.8			
No statin during follow-up							
<5%	1268	1.7 (1.6-1.8)	1.4 (1.4-1.5)	1.8	673.7	<.001	0.778 (0.771-0.786)
5% to <7.5%	495	6.2 (5.7-6.7)	5.2 (4.7-5.7)	6.1			
7.5% to <10%	369	8.3 (7.5-9.1)	6.9 (6.2-7.7)	8.6			
≥10%	1443	13.3 (12.6-13.9)	10.2 (9.6-10.8)	17.0			
Statin during follow-up							
<5%	491	1.3 (1.2-1.4)	1.1 (1.0-1.2)	2.6	106.4	<.001	0.703 (0.693-0.712)
5% to <7.5%	342	2.3 (2.0-2.5)	1.9 (1.7-2.2)	6.2			
7.5% to <10%	341	3.0 (2.7-3.3)	2.6 (2.3-2.9)	8.7			
≥10%	1779	6.0 (5.8-6.3)	4.8 (4.6-5.1)	16.7			

Abbreviations: PCE, pooled cohort equations; PREVENT, Predicting Risk of Cardiovascular Disease Events; PY, person-years.

^a^ Adjusted 10-year risk was estimated accounting for noncoronary heart disease mortality as a competing event, using the Fine-Gray subdistribution model.

In contrast, patients exposed to statin therapy during follow-up consistently had lower observed rates than estimated across all risk categories. A similar pattern was observed when using the PREVENT full equations (eTable 1 in Supplement 1). In terms of discrimination, the C statistic was 0.723 (95% CI, 0.716-0.729) for the PREVENT base model, 0.723 (95% CI, 0.717-0.729) for the PREVENT full model, and 0.725 (95% CI, 0.719-0.731) for the PCE model.

**Figure 3.**
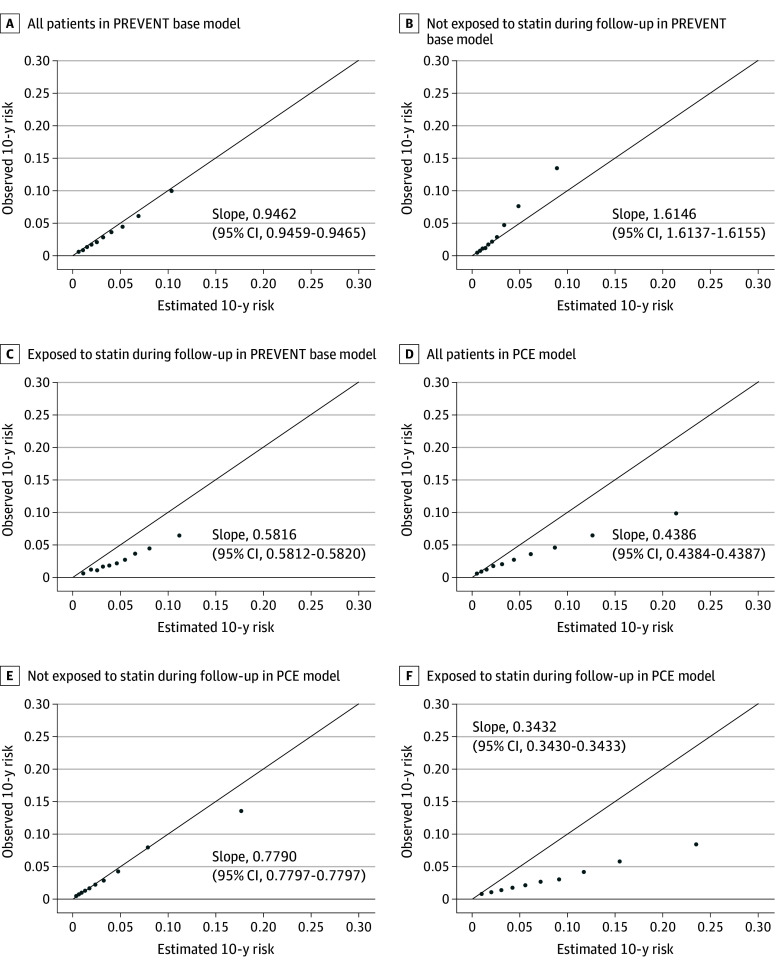
Calibration Plots Comparing Observed 10-Year Risk of Atherosclerotic Cardiovascular Events With That Estimated by the Predicting Risk of Cardiovascular Disease Events (PREVENT) Base Equations or the Pooled Cohort Equation (PCE) Within Deciles

Figure 3 shows the calibration plots comparing observed and estimated ASCVD events using the PREVENT base equations. In the overall population, the PREVENT base equations closely approximated the observed risk across all risk categories (Figure 3A). However, among patients not exposed to statin therapy during follow-up, the PREVENT base equations yielded estimated values lower than the observed outcomes (slope, 1.6146; 95% CI, 1.6137-1.6155). The PREVENT full equation also underestimated ASCVD risk in the group of patients not exposed to statin therapy (slope, 2.0416; 95% CI, 2.0404-2.0427) (eFigure 4 in Supplement 1).

PCE overestimated risk in the overall population (Figure 3D). Among patients not exposed to statin therapy, the PCE-estimated risk closely approximated their observed ASCVD risk in most deciles, except in the highest risk decile where PCE overestimated risk (Figure 3E). The PREVENT equations reclassified patients, both those who experienced an event and those who did not, into lower risk categories relative to the PCE (eTable 2 in Supplement 1).

## Discussion

In this large population-based cohort study in the US, both the PCE and PREVENT models demonstrated good discrimination in estimating 10-year ASCVD events. However, notable differences emerged when evaluating performance across key subgroups, particularly among individuals who were not exposed to statin therapy. In the statin-untreated group, the PREVENT model underestimated ASCVD risk, while the PCE model more closely approximated the observed risk. Compared with the PREVENT estimates, the PCE estimates more closely reflected what a patient’s risk would be if they never started statin therapy, an important consideration when using risk prediction equations to guide statin initiation decisions.

The American College of Cardiology Cholesterol Clinical Practice Guidelines recommend using a validated risk prediction tool to estimate 10-year ASCVD risk as the first step in considering treatment options for primary prevention.^3^ Under the current guidelines, patients with an estimated 10-year ASCVD risk between 5% and less than 7.5% are classified as having borderline risk, where statin therapy is considered if additional risk-enhancing factors are present. Patients with an estimated 10-year risk between 7.5% and less than 20% fall into the intermediate risk category, where initiation of moderate- to high-intensity statin therapy is recommended.

Given the significant treatment implications of ASCVD risk scores, it is crucial to validate these models across contemporary large populations to ensure their accuracy and reliability in guiding clinical decision-making. This study differs from other validation efforts in several key respects. First, the cohort was drawn from an integrated health care delivery system with complete capture of clinical events through hospitalization records and insurance claims, minimizing concerns about incomplete event ascertainment.^14^ Second, prior validation studies based on patient research registries may have some limitations in generalizability, since individuals who voluntarily participate in research studies may not be fully representative of the broader population.^4,15^ This study’s use of an integrated health system cohort includes a broad population of unselected individuals, increasing the applicability of findings to clinical settings. Third, all patients in this study had full 10-year follow-up data, which enhances the reliability of outcome assessment without requiring extrapolation or assumptions about long-term risk.

Our findings are consistent with previous reports indicating that PCE tends to overestimate ASCVD risk in contemporary populations.^4,6,14,15^ The extent of overestimation observed in this study is comparable to that seen in other large multiethnic cohorts, such as the Multi-Ethnic Study of Atherosclerosis cohort^4^ and the Reasons for Geographic and Racial Differences in Stroke cohort.^16^ One proposed explanation for this overestimation is the increased use of preventive therapies over the past few decades, leading to a decline in ASCVD event rates.^16^ The PREVENT equations were developed using more recent datasets to recalibrate risk assessments. A recent external validation study using data from the National Health and Nutrition Examination Survey (NHANES) found that the PREVENT model demonstrated moderate underfitting while the PCE model was overfit.^17^

In our study, the C statistic was 0.72 for PREVENT and 0.73 for PCE. These values are consistent with those reported in other studies and support the ability of both models to stratify patients by relative risk.^8,18^

For a risk stratification tool to be effective at the individual level, it should provide estimates of both untreated risk and the potential risk reduction with therapy. Evaluating the performance of PCE and PREVENT in patients who were not exposed to statin therapy during follow-up is particularly important, as these models are often used at the point of care to help guide treatment decisions. This study found that among patients not treated with statins, both the PREVENT base and full equations underestimated risk, with some individuals classified as low risk experiencing higher-than-expected event rates.

These findings suggest that a more nuanced approach may be warranted when using these equations to estimate cardiovascular risk. In general, the PCE provided the most conservative estimate of a patient’s 10-year risk, assuming no statin therapy is used during follow-up. In contrast, the PREVENT equations yielded substantially lower estimates that more closely reflected outcomes in patients receiving contemporary therapy, including statins. For individuals not receiving medical therapy during follow-up, the PREVENT-estimated risk may be considerably lower than the risk actually observed. Relying solely on the PREVENT equations to guide statin initiation could lead to undertreatment, potentially leaving individuals at high risk without appropriate preventive therapy. In comparison, using the PCE for statin initiation decisions would likely result in more patients receiving statins and reduce the chance of missing high-risk individuals who could benefit from treatment.

From a practical standpoint, clinicians using the PCE should recognize that the actual event rates may be lower than estimated if patients adhere to risk-reducing interventions. There is an evolving body of literature emphasizing the importance of accounting for treatment in clinical risk prediction models. Models that can estimate baseline risk in the absence of therapy and then project risk trajectories after the initiation of statin therapy would be particularly valuable for informing clinical decision-making at the point of care.^19,20,21^

### Limitations

Several limitations of our study should be acknowledged. First, this study is an observational retrospective analysis and therefore subject to potential biases. Outcome misclassification and underadjudication of events are possible; however, the use of multiple data sources, including claims data within an integrated health system, likely minimizes these risks. Second, while we accounted for statin use during follow-up, the decision to initiate statin therapy was not randomized. Patients who initiated statins likely differed from those who did not in ways beyond baseline risk factors and may have risk-enhancing factors not captured by the risk scores. Third, the study population was drawn from a health care system in California. While demographically diverse, it may not fully represent populations in other geographic regions, especially on a global scale. Fourth, all individuals in the study had health insurance and access to health care, which may limit the generalizability of the findings to population with limited health care access. Fifth, the study focused on 10-year ASCVD risk, while the PREVENT equations also provide a 30-year risk estimation, which was not evaluated in this study.

## Conclusions

This retrospective cohort study provides a contemporary assessment of both the PREVENT and PCE models in a large, diverse US population with complete 10-year follow-up. The PREVENT model offers advantages by incorporating social determinants of health and removing race as a factor. Risk estimates from PREVENT tend to be lower than those from the PCE and lower than the observed risk in patients not treated with statins. When used to guide statin initiation decisions, relying on PREVENT alone to estimate what a patient’s 10-year risk would be without statin therapy may lead to undertreatment.

## Notes

Supplement 1. **eFigure 1.** Cohort assembly **eFigure 2.** Kaplan-Meier curves stratified by predicted risk of cardiovascular disease events in patients with high adherence to statin therapy during follow-up **eFigure 3.** Observed and predicted incidence rates of atherosclerotic cardiovascular events among patients with high adherence to statin therapy. **eFigure 4.** Calibration plots comparing observed 10-year risk of ASCVD with that predicted by the PREVENT full equations within deciles **eTable 1.** Observed and predicted incidence rates of atherosclerotic cardiovascular events stratified by 10-year predicted risk using the PREVENT full equations **eTable 2.** Reclassification table for the overall cohort, the statin-exposed patients, and the patients not exposed to statin therapy

Supplement 2.
Data Sharing Statement
