# Supplementary material for: PREVENT and PCE Models for Estimating ASCVD Risk Stratified by Statin Exposure
Source: JAMA Netw Open. 2025 Sep 16;8(9):e2532164. doi: 10.1001/jamanetworkopen.2025.32164 (PMC12441873; doi:10.1001/jamanetworkopen.2025.32164)
Supplement: Supplement 1. — eFigure 1. Cohort assembly eFigure 2. Kaplan-Meier curves stratified by predicted risk of cardiovascular disease events in patients with high adherence to statin therapy during follow-up eFigure 3. Observed and predicted incidence rates of atherosclerotic cardiovascular events among patients with high adherence to statin therapy. eFigure 4. Calibration plots comparing observed 10-year risk of ASCVD with that predicted by the PREVENT full equations within deciles eTable 1. Observed and predicted incidence rates of atherosclerotic cardiovascular events stratified by 10-year predicted risk using the PREVENT full equations eTable 2. Reclassification table for the overall cohort, the statin-exposed patients, and the patients not exposed to statin therapy [file jamanetwopen-e2532164-s001.pdf]

Supplemental Online Content

Lee M, Onwuzurike J, Wu Y, Palmer-Toy DE, An J, Chen W. PREVENT and PCE models for ASCVD risk by statin exposure. *JAMA Netw Open*. 2025;8(9):e2532164. doi:10.1001/jamanetworkopen.2025.32164

eFigure 1. Cohort assembly
eFigure 2. Kaplan-Meier curves stratified by predicted risk of cardiovascular disease events in patients with high adherence to statin therapy during follow-up
eFigure 3. Observed and predicted incidence rates of atherosclerotic cardiovascular events among patients with high adherence to statin therapy.
eFigure 4. Calibration plots comparing observed 10-year risk of ASCVD with that predicted by the PREVENT full equations within deciles
eTable 1. Observed and predicted incidence rates of atherosclerotic cardiovascular events stratified by 10-year predicted risk using the PREVENT full equations
eTable 2. Reclassification table for the overall cohort, the statin-exposed patients, and the patients not exposed to statin therapy

This supplemental material has been provided by the authors to give readers additional information about their work.

© 2025 Lee M et al. *JAMA Network Open*.

Supplemental eFigure 1. Cohort Assembly

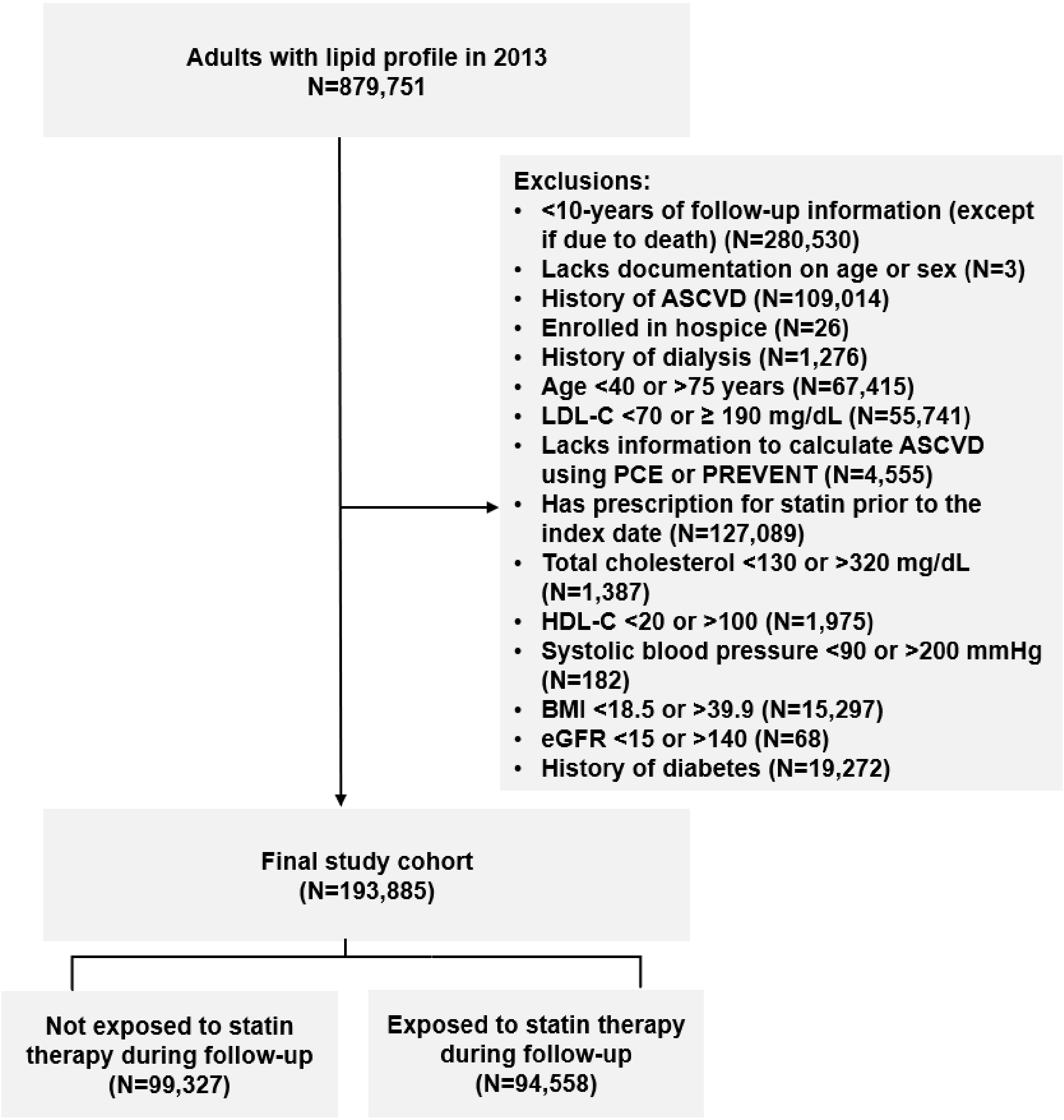

© 2025 Lee M et al. *JAMA Network Open*.

Supplemental eFigure 2. Kaplan-Meier curves stratified by predicted risk of cardiovascular disease events in patients with high adherence to statin therapy during follow-up (log rank p<0.001).

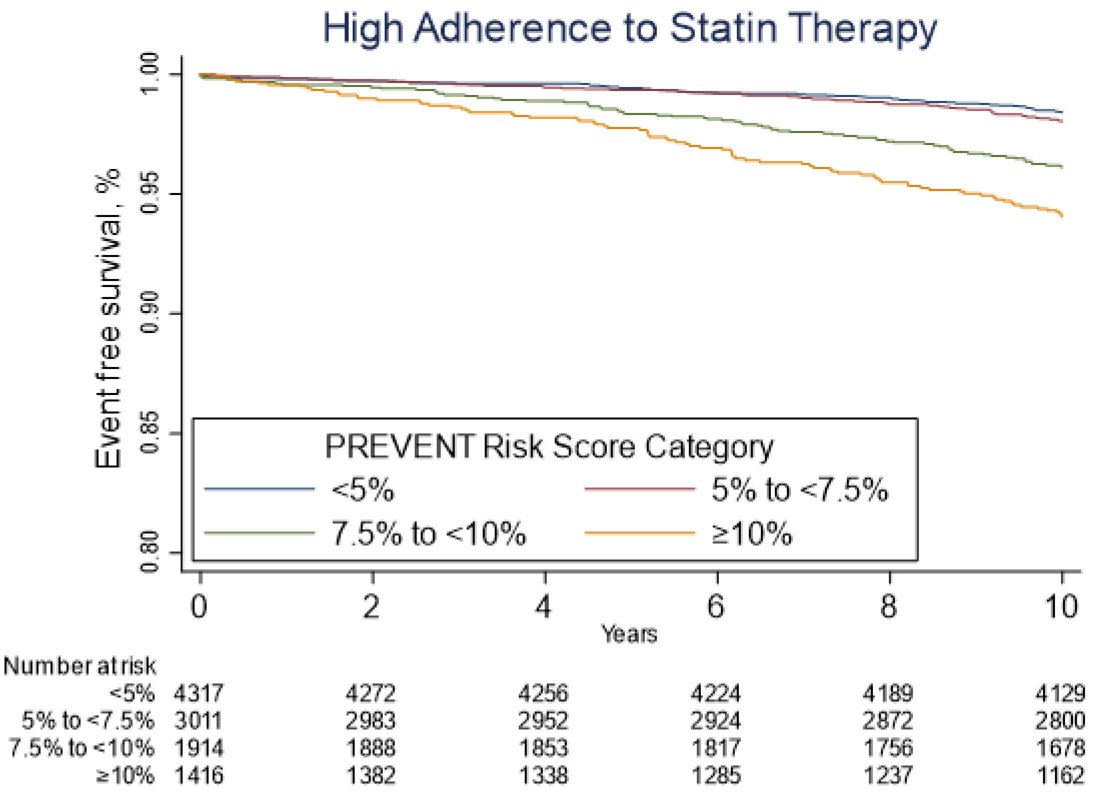

© 2025 Lee M et al. *JAMA Network Open*.

Supplemental eFigure 3. Observed and Predicted Incidence Rates of Atherosclerotic Cardiovascular Events Among Patients with High Adherence to Statin Therapy.

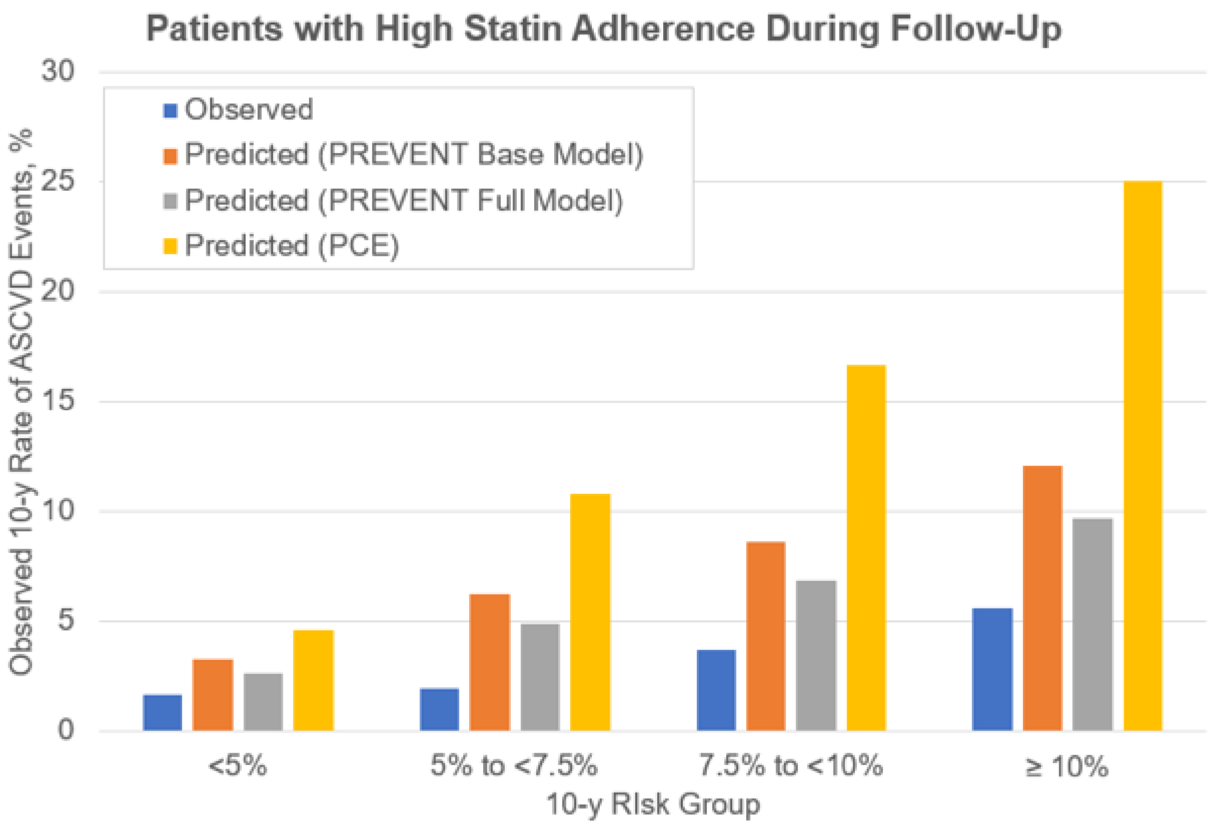

© 2025 Lee M et al. *JAMA Network Open*.

Supplemental eFigure 4. Calibration plots comparing observed 10-year risk of ASCVD with that predicted by the PREVENT full equations within deciles. (A) Plots with PREVENT full equations applied to all patients [slope (95% CI) = 1.1581 (1.1579-1.1582)]. (B) Patients not exposed to statin therapy during follow-up [slope (95% CI) = 2.0416 (2.0404-2.0427)]. (C) Patients exposed to statin therapy during follow-up [slope (95% CI) = 0.6884 (0.6880-0.6888)].

A.

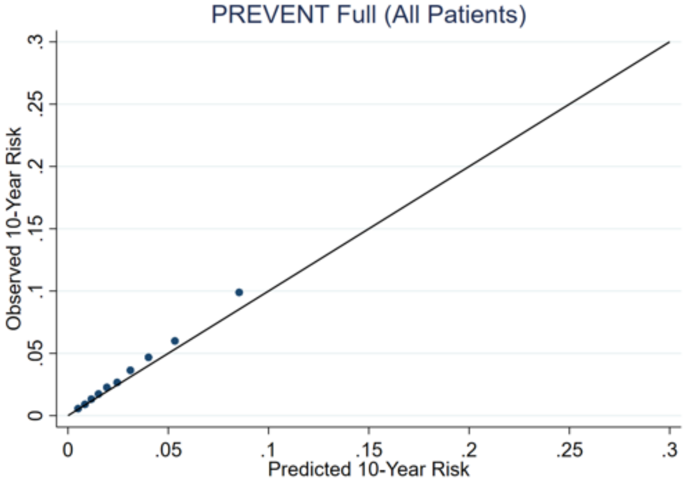

B.

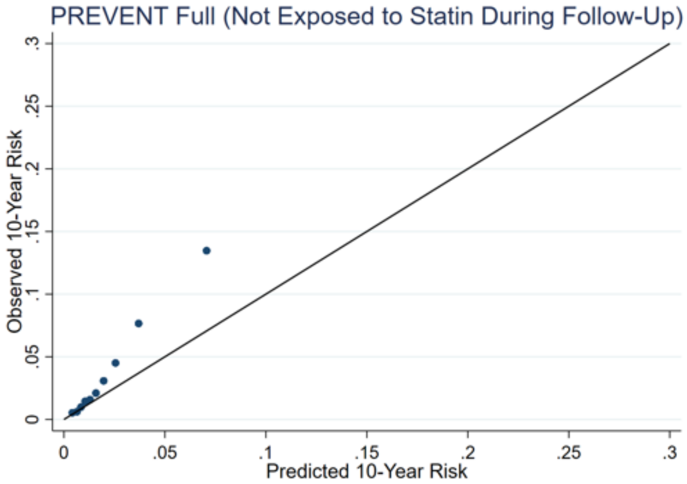

C.

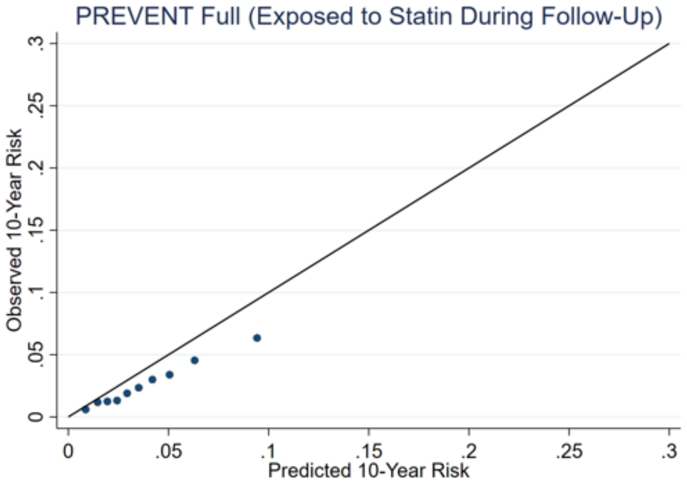

© 2025 Lee M et al. *JAMA Network Open*.

Supplemental eTable 1. Observed and Predicted Incidence Rates of Atherosclerotic Cardiovascular Events stratified by 10-Year Predicted risk using the PREVENT full equations

		10-year incidence rate / 100 person-years			Calibration		
**Estimated 10-year Risk**	**Events, n**	**Crude observed 10-year Incidence Rate/100 person-years, (95% CI)**	**Adjusted observed 10-year Incidence Rate/100 person-years, (95% CI)**	**Predicted incidence rate**	**Hosmer-Lemeshow χ^2**	**P value**	**Discrimination, C-index (95% CI)**
**PREVENT full equations**							
**All patients**							
**<5%**	3756	2.3 (2.2-2.4)	2.0 (1.9-2.1)	2.1	462.1	<0.001	0.723 (0.717-0.729)
**5% to <7.5%**	1499	7.0 (6.7-7.4)	5.6 (5.3-5.9)	6.1			
**7.5% to <10%**	801	10.2 (9.5-10.9)	7.6 (7.1-8.2)	8.5			
**≥ 10%**	472	13.3 (12.2-14.4)	9.3 (8.4-10.3)	12.4			
**Patients not exposed to statin during follow-up**							
**<5%**	2359	2.6 (2.5-2.7)	2.2 (2.1-2.3)	1.6	544.8	<0.001	0.777 (0.770-0.785)
**5% to <7.5%**	645	11.8 (10.9-12.6)	9.1 (8.4-9.9)	6.1			
**7.5% to <10%**	349	16.3 (14.7-17.9)	12.0 (10.7-13.4)	8.5			
**≥ 10%**	222	22.0 (19.4-24.6)	14.4 (12.2-16.6)	12.2			
**Patients exposed to statin during follow-up**							
**<5%**	1397	2.0 (1.9-2.1)	1.7 (1.6-1.8)	2.6	149.1	<0.001	0.701 (0.691-0.710)
**5% to <7.5%**	854	5.4 (5.1-5.8)	4.4 (4.1-4.7)	6.1			
**7.5% to <10%**	452	7.9 (7.2-8.6)	6.0 (5.4-6.6)	8.5			
**≥ 10%**	250	9.8 (6.7-11.0)	7.3 (6.4-8.4)	12.5			

© 2025 Lee M et al. *JAMA Network Open*.

Supplemental eTable2. Reclassification table for the overall cohort, the statin-exposed patients, and the patients not exposed to statin therapy.

	**10-year Risk estimated by PREVENT**				
**10-year Risk estimated by PCE**	<5%	5% to <7.5%	7.5% to <10%	≥ 10%	Total Counts
**Overall Cohort**					
**Patients who experienced an Event**					
**<5%**	1750 (99.5)	8 (0.5)	1 (0.1)	0	1759
**5% to <7.5%**	696 (83.2)	133 (15.9)	8 (0.9)	0	837
**7.5% to <10%**	328 (46.2)	370 (52.1)	9 (1.3)	3 (0.4)	710
**≥ 10%**	47 (1.5)	968 (30.0)	1153 (35.8)	1054 (32.7)	3222
**Total Count**	2821	1479	1171	1057	6528
**Patients who did not experience an Event**					
**<5%**	112707 (99.8)	230 (0.2)	6 (0.01)	2 (0)	112945
**5% to <7.5%**	18577 (83.4)	3668 (16.5)	34 (0.2)	3 (0.01)	22282
**7.5% to <10%**	7219 (48.0)	7628 (50.7)	184 (1.2)	10 (0.07)	15041
**≥ 10%**	655 (1.8)	15312 (41.3)	13114 (35.4)	8008 (21.6)	37089
**Total Count**	139158	26838	13338	8023	187357
**Patients exposed to statin therapy**					
**Patients who experienced an Event**					
**<5%**	487 (99.2)	3 (0.6)	1 (0.2)	0	491
**5% to <7.5%**	273 (79.8)	66 (19.3)	3 (0.9)	0	342
**7.5% to <10%**	134 (39.3)	200 (58.7)	5 (1.5)	2 (0.6)	341
**≥ 10%**	20 (1.1)	524 (29.5)	637 (35.8)	598 (33.6)	1779
**Total Count**	914	793	646	600	2953
**Patients who did not experience an Event**					
**<5%**	38088 (99.6)	139 (0.4)	4 (0.01)	1 (0)	38232
**5% to <7.5%**	12063 (81.7)	2684 (18.2)	23 (0.2)	1 (0)	14771
**7.5% to <10%**	5034 (45.9)	5777 (52.8)	133 (1.2)	5 (0.05)	10949
**≥ 10%**	475 (1.7)	11503 (41.6)	9810 (35.5)	5865 (21.2)	27653
**Total Count**	55660	20103	9970	5872	91605
**Patients not exposed to statin**					
**Patients who experienced an Event**					
**<5%**	1263 (99.6)	5 (0.4)	0	0	1268

© 2025 Lee M et al. *JAMA Network Open*.

5% to <7.5%	423 (85.5)	67 (13.5)	5 (1.0)	0	495
7.5% to <10%	194 (52.6)	170 (46.1)	4 (1.1)	1 (0.3)	369
≥ 10%	27 (1.9)	444 (30.8)	516 (35.8)	454 (31.6)_	1443
**Total Count**	1907	686	525	457	3575
**Patients who did not experience an Event**					
<5%	74619 (99.9)	91 (0.1)	2 (0)	1 (0)	74713
5% to <7.5%	6514 (86.7)	984 (13.1)	11 (0.2)	2 (0.03)	7511
7.5% to <10%	2185 (53.4)	1851 (45.2)	51 (1.3)	5 (0.1)	4092
≥ 10%	180 (1.9)	3809 (40.4)	3304 (35.0)	2143 (22.7)	9436
**Total Count**	83498	6735	3368	2151	95752

© 2025 Lee M et al. *JAMA Network Open*.
