# Supplementary material for: PREVENT and PCE Models for Estimating ASCVD Risk Stratified by Statin Exposure
Source: JAMA Netw Open. 2025 Sep 16;8(9):e2532164. doi: 10.1001/jamanetworkopen.2025.32164 (PMC12441873; doi:10.1001/jamanetworkopen.2025.32164)
Supplement: Supplement 2. — Data Sharing Statement [file jamanetwopen-e2532164-s002.pdf]

Data Sharing Statement

Lee. PREVENT and PCE Models for ASCVD Prediction by Statin Exposure. *JAMA Netw Open*. Published September 16, 2025. doi:10.1001/jamanetworkopen.2025.32164

Data
Data available: No

Additional Information
Explanation for why data not available: The datasets generated and analyzed are not publicly available due to their being the property of Kaiser Foundation Health Plan, Inc., but are available to interested collaborators in the context of a formal collaboration approved by the Kaiser Permanente Southern California Institutional Review Board for the Protection of Human Subjects.
